# Mouse genomic and cellular annotations

**DOI:** 10.1007/s00335-021-09936-7

**Published:** 2022-02-05

**Authors:** Helen Long, Richard Reeves, Michelle M. Simon

**Affiliations:** 1 MRC Harwell Institute, Mammalian Genetics Unit, Harwell Campus, Oxfordshire, OX11 0RD UK; 2 Nuffield Department of Medicine, University of Oxford, Oxford, UK

## Abstract

Mice have emerged as one of the most popular and valuable model organisms in the research of human biology. This is due to their genetic and physiological similarity to humans, short generation times, availability of genetically homologous inbred strains, and relatively easy laboratory maintenance. Therefore, following the release of the initial human reference genome, the generation of the mouse reference genome was prioritised and represented an important scientific resource for the mouse genetics community. In 2002, the Mouse Genome Sequencing Consortium published an initial draft of the mouse reference genome which contained ~ 96% of the euchromatic genome of female C57BL/6 J mice. Almost two decades on from the publication of the initial draft, sequencing efforts have continued to increase the completeness and accuracy of the C57BL/6 J reference genome alongside advances in genome annotation. Additionally new sequencing technologies have provided a wealth of data that has added to the repertoire of annotations associated with traditional genomic annotations. Including but not limited to advances in regulatory elements, the 3D genome and individual cellular states. In this review we focus on the reference genome C57BL/6 J and summarise the different aspects of genomic and cellular annotations, as well as their relevance to mouse genetic research. We denote a genomic annotation as a functional unit of the genome. Cellular annotations are annotations of cell type or state, defined by the transcriptomic expression profile of a cell. Due to the wide-ranging number and diversity of annotations describing the mouse genome, we focus on gene, repeat and regulatory element annotation as well as two relatively new technologies; 3D genome architecture and single-cell sequencing outlining their utility in genetic research and their current challenges.

## Established annotations in the mouse reference genome

Good annotations are reliant on the accuracy and high quality of the reference genome assembly. The Genome Reference Consortium is responsible for building, improving and providing the mouse genome assembly to the scientific community. For example a recent and major assembly release, GRCm39, saw a change in chromosome coordinates with 100s of issues resolved. This was the first major release for nine years although the consortium continually produces improvements with minor releases. Once a release is completed it is annotated by GENCODE and RefSeq (Frankish et al. 2021; O’Leary et al. 2016).

The GENCODE resource is based at the European Bioinformatics Institute (EBI-EBML). Where its goal is the '*description of all non-redundant transcripts associated with protein-coding genes and non-coding RNAs (small and long), along with the identification of all pseudogenes.*'(Frankish et al. 2019) (Table 1). The GENCODE annotation process includes manual annotation from ‘Ensembl Human and Vertebrate Analysis and Annotation’ (HAVANA) and computational annotations produced by the Ensembl gene build team. The resulting Ensembl/GENCODE geneset forms the basis of the Ensembl genome browser resource (Howe et al. 2021; Frankish et al. 2021). Both the computational and manual annotations are held in a single database where manual annotators curate the entries by approving, updating or removing computationally annotated models. Novel genes are then assigned their Ensembl stable IDs (ENSX). New technologies such as Long-read transcriptomic sequencing; Pacific Biosciences (PacBio) and Oxford Nanopore Technologies (ONT) are now utilised in their manual and automated annotation workflows where they look for evidence of putative transcripts and RNA-seq supported introns. In addition GENCODE uses many other external resources and technologies to improve their annotations especially those with low or weak support, including UniProt (UniProt Consortium 2019), APPRIS (Rodriguez et al. 2018), PhyloCSF (Lin et al. 2011), Ensembl gene trees (Yates et al. 2020), mass spectrometry and variation data (Frankish et al. 2021).

**Table 1:** RefSeq and GENCODE Established Annotations

Feature	Function / definition
Small cytoplasmic RNA (scRNA)	Small RNAs located in the cytoplasm
Ribosomal RNA (rRNA)	Non-coding RNAs that aid translation of messenger RNA to protein
Misc RNA	RNAs that cannot be denoted by other RNA classes/biotypes
Small nuclear RNA (snRNA)	Small RNA molecules, on average 150 bases long, found in the nucleus
Small nucleolar RNA (snoRNA)	Non-coding RNAs located in the nucleolus that modify other RNAs—mainly ribosomal RNAs
MicroRNA (miRNA)	Single stranded non-coding RNA elements that regulate gene expression
Long non coding RNA (LncRNA)	RNAs longer than 200 nucleotides that are not translated into functional proteins
All Pseudogenes	Mutated or deactivated sequences that mirror genes but lack introns and other sequences
Protein-coding gene	A functional unit of heredity, which contributes to a function or a phenotype
Signal recognition particle RNA (srpRNA)	RNAs located in the cytoplasm that aid the signal recognition particle complex by targeting proteins
Transer RNA (tRNA)	Transfer RNAs are highly abundant RNAs ~ 70–100 bases in length that aid in translation
Small nuclear RNA (snRNA)	Small RNA molecules found in splicing speckes and cajal bodies within the nucleus. They are ~ 150 nucleotides in length and process pre-messenger RNA

**Fig. 1:**
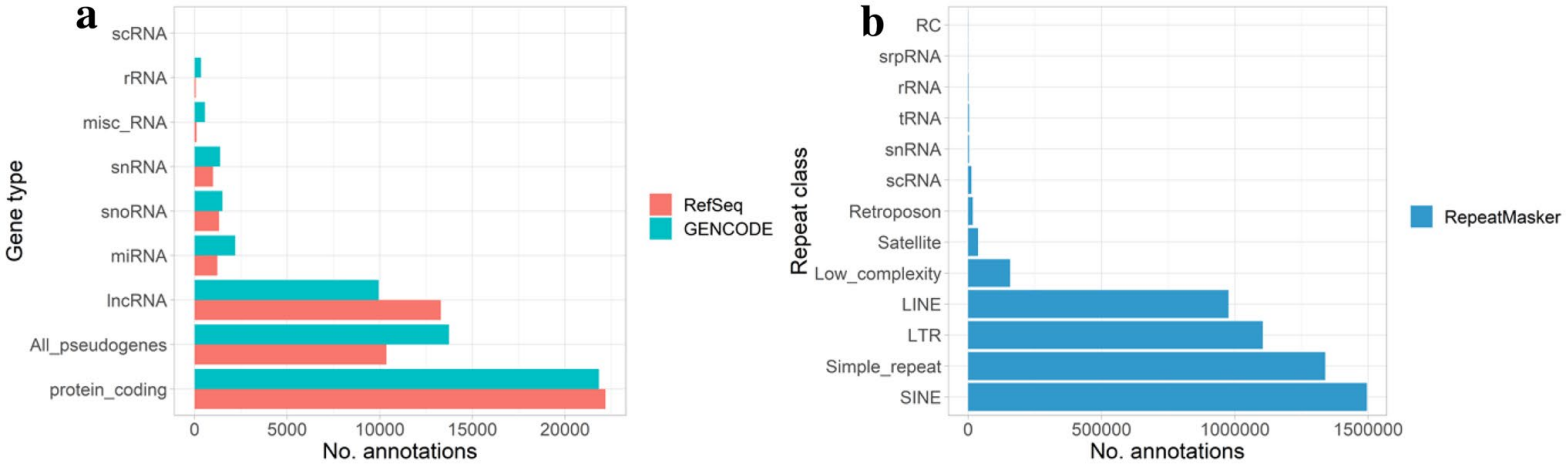
Number of annotations in: **a** GENCODE and RefSeq for mm39. Only annotations that could be obviously matched between resources have been included. **b** RepeatMasker for mm39

RefSeq is based at the National Centre for Biotechnology Information (NCBI). The database contains genomic DNA, transcripts and proteins for a multitude of organisms including mice (O’Leary et al. 2016). It aims to provide comprehensive and non-redundant annotations of protein coding genes, pseudogenes and non coding genes (McGarvey et al. 2015) (Fig. 1a). Broadly, RefSeq annotations can be split into two categories: “known” (N) and “model” (X), and helpfully their annotation accession prefixes contain information pertaining to these categories. “Known” annotations are largely manual annotations from Genbank transcripts and have RefSeq accessions with the prefixes NM_, NR_, NP_, or NG_. Annotations which are generated based on the NCBI’s automated eukaryotic annotation pipeline (Thibaud-Nissen et al. 2013) are termed ‘Model’ annotations, these have RefSeq accessions with the prefixes XM_, XR_ and XP_ (McGarvey et al. 2015). Where NM_/XM_ refers to protein coding transcripts, NR_/XR_ refers to non-coding transcripts, NP_/XP_ refers to proteins translated from NM_/XM_ transcripts (or from a gene if no annotated transcript exists), and NG_ refers to a genomic region (O’Leary et al. 2016).

Gene annotation methods applied by NCBI/RefSeq and Ensembl/GENCODE differ. This can lead to differing annotations in the same regions between resources. In order to resolve these differences the Consensus coding sequence (CCDS) project was established. CCDS aims to produce a consensus dataset for the mouse and human genomes, of protein coding regions which have the same coding sequence coordinates between resources. In order to achieve this, expert curators from the collaborating members (including those involved with the RefSeq and GENCODE projects) review regions where protein coding annotations differ between resources and attempt to reach a consensus annotation. Consensus protein coding regions are identified by stable tracked IDs which can be accessed via the CCDS web browser, FTP and other resources such as Ensembl. Expert curators also continually review existing CCDS IDs which have been flagged by quality assurance tests, curators, or users, ensuring the quality of the resource (Pruitt et al. 2009; Pujar et al. 2018). The most recent mouse CCDS release (release 23) contains 27,219 CCDS IDs corresponding to 20,484 gene IDs.

Annotations of repetitive genomic elements are performed externally from the typical annotation pipeline that annotates the mouse genome. They are commonly annotated and masked using RepeatMasker (Smit, Hubley, and Green 2013–2015). The RepeatMasker software screens for interspersed repeats and low complexity regions within the input DNA. To do this repeatmasker uses the annotations within either the Repbase library from the Genetic Information Research Institute (GIRA) (Jurka 2000) or Dfam (Storer et al. 2021) which are databases of repetitive elements. For many popular research species including mouse, pregenerated RepeatMasker annotations can be downloaded from the RepeatMasker website (Smit, Hubley, and Green 2013–2015) or University of California Santa Cruz (UCSC) Table Browser (Karolchik et al. 2004). In the mouse genome, RepeatMasker annotates a large number of repeats belonging to the SINE, Simple repeats, LTRs and LINE repeat classes (Fig. 1b) (see Table 2 for definitions).

**Table 2:** RepeatMasker Established Annotations

Feature	Function / definition
Satelite	Largely repeating short elements of AT-rich non-coding DNA that form centromeres and heterochromatin
Low Complexity	Repetitve elements of low complexity
LINE	*‘Long interspersed retrotransposable elements, respectively, that invade new genomic sites using RNA intermediates.'*
Long Terminal Repeat (LTR)	Paired sequences of DNA hundreds of base pairs long that often occur after a section of protein coding sequences
Simple Repeat	Simple duplicated sets of DNA bases
SINE	*‘Short interspersed retrotransposable elements, respectively, that invade new genomic sites using RNA intermediates.'*

Two of the main mouse specific resources which benefit from the clear, concise and auditable annotations described above are the Mouse Genome Project (Keane et al. 2011) and Mouse Genome Informatics (MGI) (Bult et al. 2019). The most widely used isogenic strain is C57BL/6 J, and is the primary subject of this review; however the scientific community uses a plethora of mouse strains for their research. Due to the sequencing of different mouse strains via the Mouse Genome Project researchers are able to compare the sequence and polymorphisms underlying annotations in the C57BL/6 J genome to different mouse strains. To date sixteen different mouse strains are available via Ensembl and UCSC with more available via the Mouse Genome Project portal. Another vital portal in mouse genetics is the Mouse Genome Informatics resource (MGI) (Bult et al. 2019). MGI curates and disseminates information on mouse phenotypic characteristics, mouse strains, alleles, gene ontologies, nomenclature and gene annotations, etc. where many of these features rely on the correct annotation of the reference genome. For example, The Mouse Genome Database Nomenclature Committee provides advice and assistance in assigning new symbols and names to genes. Typically researchers will use the human readable MGI gene symbols provided by MGI to describe their gene of interest, gene symbols are typically 3–5 characters, beginning with a capital and italicised, e.g. *Atoh1*, as opposed to all capitalised for a human gene. The challenge for both these resources and others is how to incorporate other genomic and cellular annotations described below.

## Chromatin and cis-regulatory elements annotations

Regulatory elements tightly control the spatio-temporal expression of each gene, giving rise to an abundance of different cell types. They offer a critical layer of information in understanding how the same set of gene annotations, which exist in almost every cell, can give rise to complex multicellular organisms like mice and humans. This is a fast evolving field in genetics and it is important to put these annotations in context. There are two categories of regulatory elements, the first are trans-regulatory elements, which are sequences encoding transcription affecting molecules such as transcription factors, and are not the focus of this section (Wittkopp and Kalay 2011). The second is cis-regulatory elements which are genomic sequences that regulate the transcription of nearby target genes by recruiting proteins, such as those encoded by trans-regulatory elements (Wittkopp and Kalay 2011). Three common cis-regulatory elements are promoters, enhancers and boundary elements (Oudelaar and Higgs 2021). Other classes exist including silencers/repressors, however they are less characterised and are not discussed in this section (Halfon 2020; Ngan et al. 2020). Each class is commonly associated with specific chromatin modifications or protein binding profiles which can be detected via Next Generation Sequencing (NGS) methods such as ChIP-seq. Promoters are enriched for H3K4me3 and H3K27ac histone modifications, whereas enhancers are enriched for H3K4me1 and H3K27ac histone modifications. Boundary elements are enriched for CTCF and Cohesin binding (Table 3)(Oudelaar and Higgs 2021; Wittkopp and Kalay 2011). Although these annotation classes have traditionally been thought of as distinct, there is a growing body of evidence suggesting that some regulatory elements have features typical of multiple classes (Andersson and Sandelin 2020; Oudelaar and Higgs 2021). In this section we outline resources which annotate or contextualize these annotation classes. However, it is worth noting that there are other resources, which can use different annotation classes to those outlined here e.g. ORegAnno uses “regulatory elements', not “transcription factor binding sites”, “miRNA binding sites” etc. (Lesurf et al. 2016).

**Table 3:** Regulatory annotations and resources

Feature	Function	Features	FANTOM5	Vista enhancer browser	Encode	EnhancerAtlas 2.0	3D Genome Browser	ChromHMM
*Cis-regulatory element*								
Promoter	Recruits the pre-initiation complex, located at or near the transcription start site of a gene	H3K4me3, H3K27ac	X		X			X
Enhancer	Stimulates transcription of target gene/genes, often located near target gene, however can be some distance away	H3K4me1, H3K27ac	X*	X	X	X		X
Boundary	Prevents spread of euchromatin or heterochromatin in the genome, prevents the formation of regulatory interactions between enhancers and promoters	CTCF and Cohesin binding			X			X^#^
*Primary order chromatin annotation*								
Open chromatin	Active transcription, loosely packed nucleosomes	Acetylation, ATAC-seq, DNase hypersensitivity, FAIRE-seq and MNase-seq			X			
Heterochromatin	Repressed transcription, densely packed nucleosomes. Constitutive heterochromatin found at repeats, transposons, the centromere and telomeres. Facultative heterochromatin silences genes and is developmentally regulated	H3K9me3, HP1 (Constitutive) H3K27me3, polycomb family proteins (facultative)			X			X
*Higher order chromatin annotation*								
Compartment	A and B compartments, chromatin clusters together with other chromatin from the same compartment. Commonly identified using the first two principal components of Hi-C data	‘A compartment’ enriched for genes, active transcription and open chromatin, ‘B compartment’ enriched for heterochromatin			X			
Topologically associating domain (TAD)	Self-interacting chromatin domains, thought to colocalise enhancers and their target genes, block the spread of activation/repression in the genome, and insulate genes from aberrant regulatory interactions. Identified algorithmically from Hi-C maps	Boundary elements enriched at boundaries i.e. convergently orientated CTCF binding sites			X		X	
Chromatin loop	Loop structure created by physical interaction between two DNA elements commonly within the same TAD	Boundary elements enriched at boundaries i.e. convergently orientated CTCF binding sites			X	X	X	

* = transcribed enhancers. ^#^ = annotated as insulators. Resources which either provide any annotation of that category or any example of a raw data type required to infer them have been marked with “X”

There are numerous databases which contain cis-regulatory annotations. One of which is the functional annotation of the mammalian genome project (FANTOM5) which generated Cap Analysis of Gene Expression (CAGE) seq in mouse and human cells (Lizio et al. 2015, 2019). This allowed the precise annotation of known promoter locations and the identification of new promoters, as well as annotation of promoter activity in different cell types (measured by expression level of CAGE peaks). The use of CAGE seq also enabled the detection of transcribed enhancers (Noguchi et al. 2017; Arner et al. 2015). FANTOM5 annotations are available via the FANTOM web portal as data files or promoter/transcription start site (TSS) annotations, and can be queried through the web interface: Semantic catalog of Samples, Transcription initiation And Regulators (SSTAR) (Abugessaisa et al. 2016). A second regulatory annotation resource is the Vista Enhancer Browser (Visel et al. 2007). The Vista Enhancer Browser contains mouse and human enhancers which have been experimentally validated for enhancer activity using a lacZ reporter gene in transgenic mouse embryos. The spatial expression pattern of each enhancer, as detected by lacZ staining is also provided. Currently 3231 regulatory elements have been tested and 1653 were found to have enhancer function.

A key complexity in cis-regulatory element annotation is that regulatory elements can be active or inactive in different cell types and timepoints, whilst active they regulate the transcription of genes. In order to understand the activity of a regulatory element annotation within a given cell type, it is useful to consider its context in terms of the chromatin annotations in that cell type. Chromatin annotations can be broadly split into primary order and higher order chromatin architecture annotations which are hierarchically organised in 3D space (Chang et al. 2018). Primary order chromatin architecture is less complex and is further organised into higher order structures with greater complexity. Primary order chromatin architecture refers to the level of compaction/accessibility of chromatin caused by the nucleosome density (Chang et al. 2018). Heterochromatin is tightly packed and genes within it are transcriptionally inactive (Vignaux, Bregio, and Hathaway 2019; Murakami 2013; Saksouk et al. 2015; Libbrecht et al. 2019). Conversely, euchromatin is loosely packed and contains actively transcribed genes (Vignaux, Bregio, and Hathaway 2019) (Table 3). Therefore, cis-regulatory elements falling within a euchromatin annotation may be more likely to be active. The primary order chromatin structure of a cell type can be profiled and annotated using techniques such as ATAC-seq, DNase hypersensitivity, FAIRE-seq and MNase-seq (Chang et al. 2018). Higher order chromatin architecture annotations refer to loops, topologically associating domains (TADs) and compartments. Cis-regulatory elements are largely thought to impact their target genes by physically interacting with them, forming structures known as a loops (Yu and Ren 2017). This means that loop annotations can be used as direct evidence to link enhancers and target genes. The majority of loops fall within larger chromatin structures known as TADs (Dixon et al. 2012; Nora et al. 2012). TADs are sections of the genome which preferentially interact with themselves in 3D space. They are thought to colocalise cis-regulatory elements and their target genes and have insulators at their boundaries which reduce inter-TAD interactions (Dixon et al. 2012, 2016). This means TAD boundary annotations can often narrow down the possible candidate target genes of an enhancer. TADs are then further organised within two chromatin structures known as the A and B compartments, where the A compartment is highly enriched for euchromatin and the B compartment is highly enriched for heterochromatin (Lieberman-Aiden et al. 2009). Compartment identity annotations of cis-regulatory elements can help to inform which regulatory elements are likely to be active in a given cell type or developmental timepoint. Higher order chromatin structures are commonly profiled and annotated using chromatin conformation capture (3C) techniques (Dekker et al. 2002) e.g. Hi-C (Lieberman-Aiden et al. 2009) or ligation free methods such as GAM (Beagrie et al. 2017) or SPRITE (Quinodoz et al. 2018).

There are several resources which provide cis-regulatory annotations along with primary and/or higher order chromatin architecture annotations. One of the most popular is the Encyclopedia of DNA Elements project (ENCODE) (Davis et al. 2018), which has coordinated an effort to generate datasets to analyse primary order and higher order chromatin architecture, transcription, DNA methylation, histone modification, transcription factor occupancy and RNA binding proteins, in different cell/tissue types from model organisms including mouse. ENCODE datasets have been generated using standardised pipelines which ensure consistency between datasets. All data is accessible via the ENCODE portal, making ENCODE an invaluable resource for identifing and/or characterising the function of cis-regulatory elements in a particular cell type. Additionally the ENCODE data has been used to develop a registry of candidate cis-regulatory elements which currently includes 339,815 mouse annotations covering 3.4% of the mouse genome. These annotations can be accessed using a web-based server called SCREEN (ENCODE Project Consortium et al. 2020). The ENCODE dataset has also been utilised by Ensembl to create the mouse Ensembl regulatory build which can be accessed via the Ensembl web browser (Howe et al. 2021; Zerbino et al. 2015).

In addition to large scale data generation efforts such as ENCODE there is a wealth of publicly available data in online repositories such as Gene Expression Omnibus (GEO) that can be downloaded and used to identify cis-regulatory elements (e.g. chromatin modification or protein binding profiles) or contextualise them (e.g. primary or higher order chromatin assays) (Barrett et al. 2013). EnhancerAtlas 2.0 has utilised 16,055 data sets from GEO (Barrett et al. 2013), ENCODE (Davis et al. 2018), FANTOM5 (Andersson et al. 2014) and the Epigenome Roadmap (Roadmap Epigenomics Consortium et al. 2015) to annotate 13,494,603 enhancers in nine species including mouse. Enhancers are predicted for each cell type using an unsupervised learning approach and their target genes are predicted using an algorithm called Enhancer And Gene based Learning Ensemble (EAGLE) (in which 3C based methods e.g. Hi-C are used as the training data). All enhancer predictions and target genes can be downloaded from the web based portal (Gao and Qian 2020).

In addition to resources containing both cis-regulatory annotations and higher/primary order chromatin architecture annotations, there are now dedicated resources containing only higher order chromatin architecture annotations. A popular database containing TAD and loop annotations for mouse tissues is the 3D Genome Browser (Wang et al. 2018). However, TADs are currently algorithmically defined and several studies have noted huge variation in TAD annotations depending on the algorithm used (Dali and Blanchette 2017; Forcato et al. 2017; Zufferey et al. 2018). Further advances in the biological definition of TADs and the adoption of a gold standard method for their detection may increase their future utility (de Wit 2020; Eres and Gilad 2021).

Many of the resources outlined above allow users to access pre-identified/predicted enhancers and chromatin annotations for multiple cell types and tissues. However, is often desirable to use newly generated data as well as integrating other publicly available data sets to identify and contextulise cis-regulatory elements from scratch. A popular algorithm to achieve this is ChromHMM which uses a hidden markov model (HMM) method to predict chromatin states (active promoter, strong enhancer, poised enhancer etc.) from input data such as histone modifications in a given cell type (Ernst and Kellis 2017). Precomputed ChromHMM predictions are also available for some cell types in ENCODE (Davis et al. 2018) and the UCSC genome browser (Kent et al. 2002).

The resources outlined in this section provide freely available cis-regulatory element annotations and primary/higher order chromatin architecture annotations for many cell types. However cis-regulatory elements and their activity are very cell type specific, future improvements will come from the generation and integration of data from an increasingly comprehensive selection of mouse cell types and tissues. It should also be noted that several of the resources outlined here provide annotations of the same features but using differing data modalities as evidence, differing algorithmic methods and/or differing levels of confidence. Therefore, users should select the resource most appropriate to their specific study with care. The field may also benefit in the future from greater attempts to validate annotations and provide consensus between resources (similar to the protein coding gene annotations performed by the CCDS project).

## Single-cell annotations

Understanding the activity of the genomic annotations in a multicellular context has been vastly improved by the advent of single-cell methods (Eberwine et al. 1992; Tang et al. 2009; Huang 2009) and the resulting annotations. The annotations provide detailed information about the spatio-temporal activity of genomic features, such as genes and enhancers, at a cellular resolution. These annotations can be generated by multiple methods including imaging and sequencing. In this section we focus on single-cell annotations with particular emphasis on single-cell RNA sequencing (scRNA-Seq) in which the transcriptome of each cell is sequenced separately.

In order to study the activity of genomic features using single-cell data we must first annotate the cell type identity of each sequenced cell. Therefore, we define a single-cell annotation as any description of an individual feature of a cell, including cell type classification and the genes expressed. The first few mouse scRNA-Seq experiments were carried out on blood and brain tissues, this was followed by the generation of atlases of organ development and the whole mouse (Han et al. 2018; Cao et al. 2019). Despite these rapid advances, annotations for single-cell expression are still in their adolescence, with research continuing to explore new tissues. For some tissues, dissection and cellular dissociation are difficult (e.g. adipose and neuronal); therefore in order to generate single-cell annotations for these tissues alternate techniques such as single-cell nuclei sequencing are required.

**Fig. 2:**
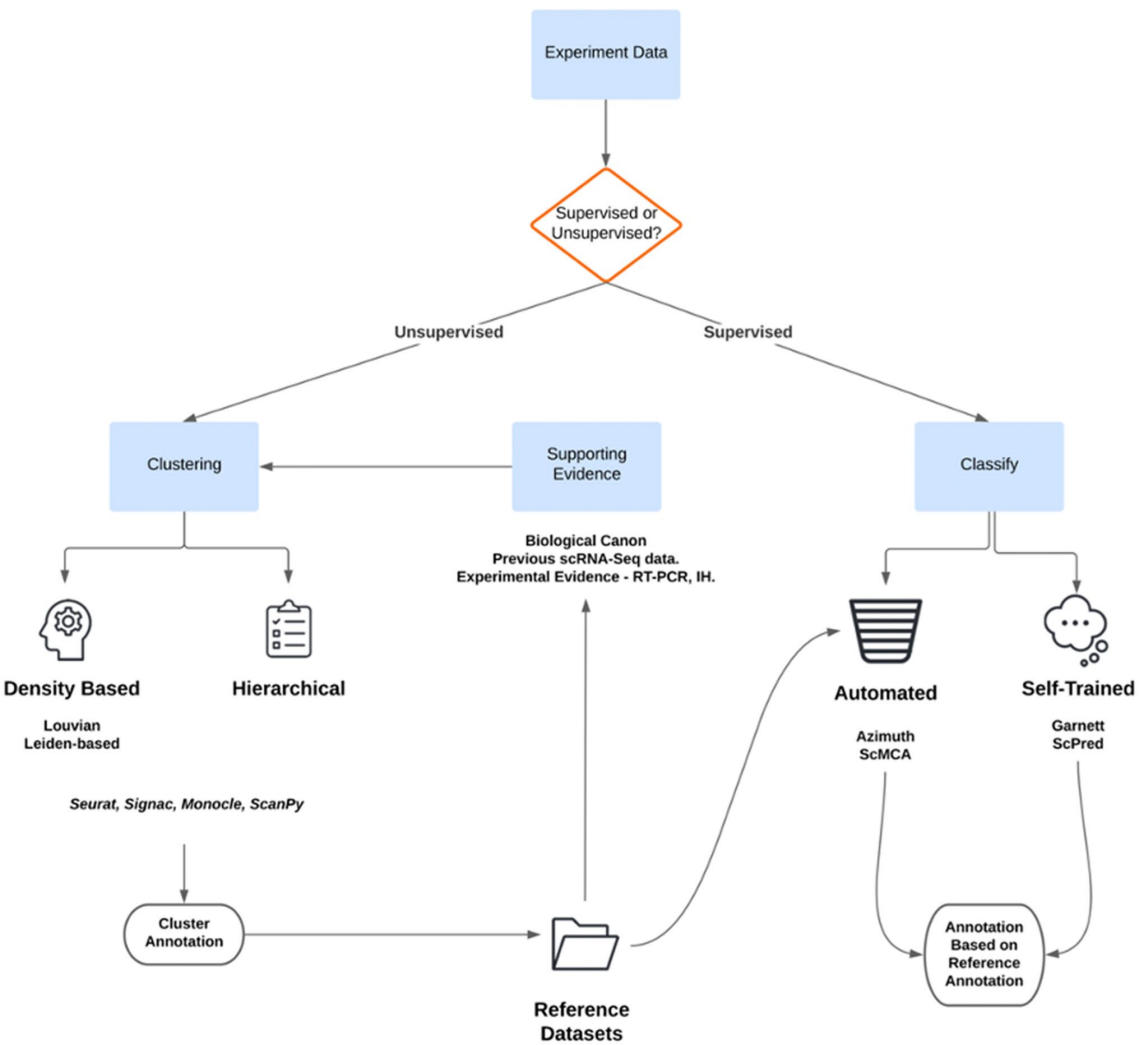
Annotation of scRNA-Seq data. **A** Single-cell experimental data is taken as input. **B** Input data is analysed using either unsupervised or supervised analysis. **C** Unsupervised analysis is done via clustering, for which there are many algorithms and single-cell tools, such as Seurat, Signac, Monocle and ScanPy **D** Clustering is done with the guidance of supporting evidence from previous data to identify known clusters, and where necessary identify novel clusters, leading to a new single-cell cluster annotation. **E** The cluster annotations then form part of the reference datasets which feed into supporting evidence, **F** and also are the basis for supervised classification of single-cell data. **G** Supervised classification of single-cell data relies on reference annotations to label cells. Some tools such as Alona and scMCA enable automated annotation, but other tools such as Garnet and ScPred are self-trained. **H** Supervised classification then produces annotated cells based off of a reference dataset of choice

The first step in single-cell annotation involves grouping single-cells with similar expression patterns together into a cluster and predicting the cell type identity of the cluster. Traditional single-cell analyses perform the clustering step using an unsupervised identification of cell types and are adept at finding novel cell types in an unbiased manner (Kiselev et al. 2019). A simple pipeline carries out sequence alignment, dimensionality reduction and feature selection (Andrews et al. 2021). Thereafter, there is a choice between a wealth of different clustering algorithms and analysis tools (Traag et al. 2019; Kiselev et al. 2017, 2019) Fig. 2). These tools produce a set of clusters, each cluster representing a cell-type (Fig. 2, pt. D). Once cells have been clustered into cell types, their cell type identity must be annotated. During cluster annotation the highest and most specifically expressed genes in each cluster are used to characterise cells. Typically this unsupervised method is accompanied by manual annotation involving either an established consensus for a cell annotation, previous scRNA-Seq, or other expression datasets e.g. Lifemap (Edgar et al. 2013) or marker identification from other literature (Fig. 2, pt F).

Alternatively clusters can be identified and annotated via supervised methods, namely classifiers, defined here as computational models used to annotate new single-cell data using annotations from previous single-cell data. (Fig. 2, pt E/G). Many classifiers have been developed e.g. Garnett (Pliner et al. 2019), Alona (Franzén and Björkegren 2020), scMCA (Sun et al. 2019), clustifyr (Fu et al. 2020) and SCPred (Alquicira-Hernandez et al. 2019). Automated classifiers such as ScMCA and Alona can reduce the computational barrier to single-cell analysis (Franzén and Björkegren 2020; Sun et al. 2019). Other supervised classifiers Garnett, SCPred and clustifyr require more expertise but allow the training of a classifier based on any reference tissue – and therefore are less reliant on what reference datasets are available. As more scRNA-Seq experiments become commonplace, the more concise and community lead single-cell cluster annotation will be. This may lead to classifiers becoming more regularly used to annotate single-cell data.

There are numerous resources which provide pre-annotated single-cell data (Table 4). However, they are currently disparately provided across many different resources, which may result in inconsistencies in cell-type annotation. Among the most easily accessible are cell browsers showing data from atlases for whole adult mouse and mouse organ development e.g. Tabular Muris which allows exploration of single-cell data in multiple mouse organs through a web browser (Han et al. 2018; Cao et al. 2019; Tabula Muris Consortium et al. 2018). Tabular Muris data is also available through the UCSC genome browser which displays the cell-type expression profile for each gene within a gene track (Kent et al. 2002). There are also single-cell databases that store and categorise single-cell datasets (GEO (Barrett et al. 2013), PangaloDB (Franzén, Gan, and Björkegren 2019), EBI expression atlas (Papatheodorou et al. 2020), Single Cell Portal—Broad Institute (https://singlecell.broadinstitute.org/single_cell). The EBI expression atlas has reanalysed all experiments present in its database; however, not all data from the original experiments are available due to quality control measures, and not all data is scRNA-Seq (traditional RNA-Seq and microarray data is also available).

**Table 4:** Resources to aid annotating a cell

Resource	Tissue availability	Metadata	Data used to build resources
Single-cell Mouse Cell Atlas scMCA	Whole Mouse Adult,	Cell Type, Tissue, Developmental Stage	scRNA-Seq
Mouse Organogenesis Cell Atlas	Whole Mouse Adult	Cell Type, Developmental Stage	scRNA-Seq
UCSC Cell Browser	Adult Mouse, Embryonic Mouse, Mouse Nervous System	Cell Type, Tissue, Developmental Stage, Tissue, Experiment Specific Data	scRNA-Seq
EBI expression Atlas	Mouse, Brain, Heart, Gonadal	Species, Cell Type, Tissue, Technology	scRNA-Seq, RNA-Seq, snRNA-Seq, Microarray, scATAC-Seq
Monocle / Garnett	Mouse Brain and Spinal Cord, Lung	Cell Type, Species, Tissue	scRNA-Seq
Pangalo DB	Brain, Intestine, Skin, Thymus, Spleen, Heart, Lung,	Cell Type, Tissue, Library Protocol, Number of Cells, Strain and or Genotype, Number of Expressed Genes, Accession number	scRNA-Seq
Alona	Brain, Bone Marrow, Skin, Epididymus and vas deferens	Cell Type, Accession Number, Species, Tissue	scRNA-Seq
Single Cell Portal Broad	Brain, Lung, Aging Mouse Brain	Species, Cell Type, Tissue, Technology, Disease, Sex, Library Protocol, Age	scRNA-Seq, scATAC-Seq
Mouse Brain Atlas	Whole Mouse Brain	Cell Type, Developmental Stage	scRNA-Seq
LifeMap	Developmental Mouse and Stem Cells	Cell Type, Anatomical Compartment, Developmental Path, Progenitor Status, Developmental Time, Number of associated Genes, Signals, High throughput, Matched Cultured Cells, Disease,	RNA-Seq, Microarray, In situ hybridisation
Allen Brain-Map	Whole Mouse Brain	Cell Type	scRNA-Seq

Table of resources available for single-cell level data, detailing the tissue types available, the metadata stored there and the modality in which the single-cell data has been captured in

scRNA-Seq data has notable technical aspects that potentially limit the ability to interpret single-cell data. The most commonly anticipated technical limitation of scRNA-Seq is the high level of dropouts. Dropouts are defined as zero values that are due to a failure to capture RNA for individual genes, specific to individual cells (Kharchenko et al. 2014). This happens commonly where there are insufficient quantities of starting RNA during sequencing. Typically, scRNA-Seq only captures a fraction of the total RNA per cell (Stegle et al. 2015; Grün et al. 2014). However, zero inflation is also potentially a reflection of biological variation (as “genes in the same pathway tend to exhibit similar dropout pattern” (Qiu 2020)), and that the proportion of zeros in the dataset can be used to inform clustering analyses (Kim et al. 2020). Qiu et al. speculate that dropouts can be as instructive as highly variable gene selection (Qiu 2020). In contrast, many tools exist to eradicate dropouts via differing methods of imputation while preserving “biologically silent” genes (Talwar et al. 2018; van Dijk et al. 2018; Ran et al. 2020). In time, increases in sequencing depth and improvements in RNA capture per cell should help alleviate these symptoms of single-cell analysis. Doublets also are a cause for concern; they can occur when two or more cells are identified as a single cell mainly due to the cell capture process on a micro-fluidics device. However, there are multiple methods to combat this, including those that model for the potential combination of cell types present in the dataset (DePasquale et al. 2019). High cell counts per sample will also help tools to distinguish doublets that appear to be due to ‘hybrid-profiles’, combinations of different cell types from genuine processing errors. The advancement of these methods will provide confidence to spurious, rare or low expression data that may be genuine biological results that occur when studying RNA splicing as well as epigenetic analysis.

There are also important technical considerations when using single-cell annotations from the literature or resources, which are often compounded by the number of different resources, the lack of consistency between resources and the lack of searchable metadata. When looking for a dataset to use as a reference, tissue specificity is the priority. Expression can change drastically within a lineage, a tissue, between strains, between species and at different time points. Therefore, finding a matching tissue or cell-type can be an issue. Power is also critical to ensure a dataset will contain cells of interest. As the number of cells increases within an experiment, the likelihood of identifying rare cell types increases. Tools such as https://satijalab.org/howmanycells/ can help identify the total number of cells necessary in a dataset to find cell types of interest. Sequencing depth is also important but should be balanced against the number of cells (Menon 2018). Lastly, the current plethora of computational tools to determine every step in the single-cell pipeline can affect the annotation, highlighting the need for a consensus workflow/pipeline.

## Conclusion and future of genomic and cellular annotations

The challenges for the future of genomic and cellular annotations are unbound and go far beyond what has been described in this review. Established annotations such as gene annotations often form the foundation of research projects. Meanwhile, chromatin and regulatory annotations provide an important layer of information widening our understanding of how these genomic annotations give rise to the complex array of cell types found in multicellular organisms. To this end, the layers of annotations discussed in this review should be integrated to provide detailed information about spatiotemporal gene regulatory networks in different cell types. Efforts are underway, some outlined here but the challenges are vast due to the high-dimensionality of the data.

Recent spatio-temporal annotations including the 3D genome and genes expressed in different single-cell types are heavily influenced by NGS datasets which are currently produced by individual labs and some large initiatives. Some less established annotation classes are not yet standardised and methods are not fully community sanctioned making the results harder to interpret. Furthermore we are rapidly moving towards annotating time course data where the current concerns may be heightened. Exciting technological advances are facilitating the addition of invaluable new classes of annotations but despite the good example set by many initiatives, we should be cautious when utilising recent annotations until they reach a level of standardisation similar to the established annotations.

In our opinion the best way to combat the challenges of annotating results from multi-omic datasets as well as aiding the standardisation of these datasets is two fold; one to develop more advanced methods to interpret the data produced by different resources/labs by the development of multivariate statistics and Machine Learning (ML) methods. And two, to develop multiple frameworks or ontologies where researchers must adopt this framework to their data prior to publication. Both these directions are underway in various aspects ranging from multi-omic atlases’ that adopt ML methods, to data repositories with compulsory rules, to new ontology developments. However a lack of consensus to the methods required for users to derive robust reproducible results, as well as the fast paced advancements in NGS will continue to make annotating these datasets challenging.
